# Proteomic Analysis of Differentially Accumulated Proteins in Cucumber (*Cucumis sativus*) Fruit Peel in Response to Pre-storage Cold Acclimation

**DOI:** 10.3389/fpls.2017.02167

**Published:** 2018-01-18

**Authors:** Bin Wang, Fei Shen, Shijiang Zhu

**Affiliations:** Guangdong Province Key Laboratory of Postharvest Physiology and Technology of Fruit and Vegetables, College of Horticulture, South China Agricultural University, Guangzhou, China

**Keywords:** cucumber fruit, cold storage, proteomic profile, defense response, chilling stress

## Abstract

Harvested fruits are still living organs and respond to environmental stimuli. Low temperature storage is effective in extending life of harvested fruit, but it may also cause chilling injury. Cold acclimation has been shown to induce chilling tolerance in plants, but what proteomic changes caused by cold acclimation are related to defense against chilling stress remains largely unclear. Here, 3 d of pre-storage cold acclimation (PsCA) at 10°C reduced chilling injury and secondary disease severity in cucumber stored at 5°C by 51 and 94%, respectively, compared with the control which was directly stored at 5°C. Proteomic analysis of cucumber peel identified 21 significant differentially-accumulated proteins (SDAPs) right after PsCA treatment and 23 after the following cold storage (PsCA+CS). These proteins are mainly related to stress response and defense (SRD), energy metabolism, protein metabolism, signal transduction, primary metabolism, and transcription. The SRD proteins, which made up 37% of the 21 and 47% of the 23, respectively, represented the largest class of SDAPs, and all but one protein were up-regulated, suggesting accumulation of proteins involved in defense response is central feature of proteomic profile changes brought about by PsCA. In fruit just after PsCA treatment, the identified SDAPs are related to responses to various stresses, including chilling, salt stress, dehydration, fungi, bacteria, insects, and DNA damage. However, after prolonged cold storage, the targeted proteins in acclimated fruit were narrowed down in scope to those involved in defense against chilling and pathogens. The change patterns at the transcription level of the majority of the up-regulated differentially-accumulated proteins were highly consistent with those at protein level. Taken all, the results suggest that the short-time cold acclimation initiated comprehensive defense responses in cucumber fruit at first, while the long term storage thereafter altered the responses more specifically to chilling. These findings add to the understanding of plants' molecular responses to cold acclimation.

## Introduction

Harvested fruit, although removed from the supporting tissues, continues as a living organ responsive to environmental stimuli. In order to fulfill its critical role in protecting seeds and guaranteeing that the seeds mature and disperse, the fruit needs to respond to environmental changes quickly and effectively. Chilling is one of the common environmental stresses that the fruit has to cope with as a reproductive organ (Valenzuela et al., 2017) or as a commercial product during storage and transport. Chilling injury in fruit would endanger the development of seeds. To combat this stress and make sure that seeds are properly matured, the fruit has evolved the ability to get acclimated at the cue of chilling temperature, a process referred to as cold acclimation, by which a plant develops cold tolerance after an initial exposure to a critical temperature (Thomashow, 1999; Wang and Zhu, 2017).

Over the years, many studies have been reported on plants' molecular responses to cold acclimation, which have especially addressed the role of CBF (CRT/DRE-binding factor) pathway in acquisition of cold tolerance. In *Arabidopsis*, the CBF regulon has been identified as a master regulator of CA-mediated chilling tolerance (Thomashow, 2010). The unified ICE (inducer of CBF expression)-CBF pathway provides a transcriptional feedback control of freezing tolerance to sustain plant development and survival during cold acclimation (Kim et al., 2015). However, cold acclimation is a complex process that includes signal transduction and regulation of transcription (Thomashow, 1999). Multiple regulatory pathways are activated during cold acclimation in addition to the CBF cold response pathway (Fowler and Thomashow, 2002). The increase in freezing tolerance that occurs with cold acclimation is only partially dependent on the CBF-CRT/DRE regulatory module (Park et al., 2015). Moreover, so far the research has mainly focused on transcriptome profile changes (Svensson et al., 2006; Doherty et al., 2009; Thomashow, 2010; Shi et al., 2015). It is necessary to investigate what happens to proteomic profile under cold-acclimated condition, since proteins are the major class of functional molecules in plant cells (Renaut et al., 2006; Chen et al., 2015).

Among the many techniques used for studying proteomic profiles, two-dimensional gel electrophoresis (2-DE) has proved a dependable approach in investigating responses of harvested fruits to environmental changes or treatments. 2-DE was used to show that heat shock proteins, cysteine proteases, and dehydrins are related to heat-induced chilling tolerance in peach (Lara et al., 2009), thioredoxin peroxidase and glycine-rich RNA binding proteins are involved in responses to cold stress in ripening tomato (Vega-García et al., 2010), and thaumatin-like proteins play roles against chilling injury in peach fruit (Dagar et al., 2010). 2-DE approach revealed that ATP synthesis, ROS scavenging, protective compounds synthesis, protein refolding and degradation were involved in ethylene-induced chilling tolerance in banana fruit (Li et al., 2015). Recently, 2-DE has been used to investigate the proteomic changes caused by cold acclimation in peaches (Tanou et al., 2017) and wheat (Gharechahi et al., 2014). However, this work only analyzed the proteomic profile after cold storage or freezing, but did not address what happened to proteomic profile right after acclimation. The prolonged cold storage or subsequent freezing could result in great changes in proteomic profile. The comparison between the proteomic profile changes right after cold acclimation and those after the following cold storage or freezing treatment could reveal key changes that may help gain insight into mechanisms of cold acclimation in enhancing chilling tolerance.

Cucumber (*Cucumis sativus* L.) (cucumber refers to harvested fruit and not to the whole plant) is a chilling-sensitive fruit. When exposed to temperature below 10°C, cucumber is subject to chilling injury, which is manifested as obvious pitting and/or dark watery patches on the peel surface (Yang et al., 2011; Liu et al., 2016). Since cold acclimation is used to enhance chilling tolerance in cucumber, it would obviously reduce the severity of chilling injury of the peels. Therefore, the peel makes good material for investigating proteomic changes connected to chilling tolerance induced by cold acclimation.

To shed light into mechanisms underlying fruit's proteomic responses to cold acclimation, a comparative proteomic analysis was performed to identify how the proteomic profile in cucumber fruit peel changes in response to pre-storage cold acclimation (PsCA). Moreover, this study compared the proteomic profile differences between the fruit just after cold acclimation and that after the following cold storage, in an attempt to reveal proteins that may contribute to defense against prolonged exposure to chilling stresses.

## Materials and methods

### Treatment and storage conditions

During the years from 2013 through 2016, experiments on effects of cold acclimation on chilling tolerance of cucumber in relation to proteomic responses were conducted in South China Agricultural University, Guangzhou City, Guangdong Province, China. Cucumber fruits (*C. sativus* L. cv Huaqing) were harvested at commercial maturity from a farm in Yinan County, Shandong Province, China. Fruits were selected based on uniform size and lack of physical injury or infection after arrival at the laboratory within 24 h of harvest. For PsCA treatment, the fruit were first incubated at 10°C for 3 d and then stored at 5°C with 80% relative humidity, with the fruit directly placed at 5°C under the same conditions as the control. During cold acclimation and storage, all treatments were put in darkness. All samples were kept in polyethylene film bags (0.03 mm in thickness). Three biological replicates, each containing at least 25 fruit, were used for each treatment.

### Chilling severity evaluation

Chilling injury indices (CII), secondary disease indices (SDI), and electrolyte leakage (EL) were assessed as described previously (Wang and Zhu, 2017). Three replicates were performed for each treatment.

CI development was observed during storage at 5 ± 1°C and secondary disease development was observed at ambient temperature (20°C). Fruit were sorted into five CI or SD severity categories. CI or SD indices were evaluated using a 0–4 scale based on the percentage of fruit surface with pitting (CI symptoms) or fruit surface with decay (SD symptoms), score 0 (no signs of surface pitting or decay), score 1 (<25%), score 2 (25–50%), score 3 (50–75%), score 4 (>75%). CI or SDI index = [(N_Y_ × Y)]/4∑N_Y_, where Y represents chilling injury or surface decay severity (0–4) and N_Y_ represents the number of cucumber fruits with the corresponding severity score. The CII or SDI index ranged from 0 to 1.

EL was evaluated during storage at 5 ± 1°C. Twenty discs of peel tissues, 5 mm in diameter and 1 mm in thickness, were excised from a single cucumber fruit with a stainless steel cork borer, with three replications. After being washed three times with double distilled water, 20 pieces were put into 25 mL of double distilled water and incubated 2 h. Electric conductivity (EC) was measured with conductance bridge (DDS-307, Leici Electron Instrument Factory, Shanghai, China). Total EC was determined after keeping the tube boiling for 30 min, and EL was expressed as percentage of total EC.

### Chlorophyll fluorescence determination of cucumber peel

The cucumber was cut into three equal parts and the middle part was used to measure chlorophyll fluorescence using a chlorophyll fluorometer (IMAG-K7, Walz, Germany). Cucumber fruit were dark-adapted for 30 min for measuring PSII quantum yield (Fv/Fm). Fv/Fm was used to express chlorophyll fluorescence parameter of the cucumber peel. Three replicates were performed for each treatment.

### Protein extraction and 2-DE separation

The extraction of total proteins was performed following the method described by Chan et al. (2007) with some modifications. Briefly, cucumber peel (5.0 g) was immediately ground in liquid nitrogen to fine powder and then homogenized in 20 mL of homogenization buffer [20 mM Tris-base (pH 7.8), 1 mM PMSF, 10% (w/v) PVP, 1% (w/v) DTT and 1% (v/v) Triton X-100]. The homogenate was centrifuged at 12,000 × g for 15 min at 4°C. The supernatant was collected and extracted with an equal volume of Tris-HCl (pH 7.8) buffered phenol for 30 min. After centrifugation, proteins were precipitated from the phenol phase with 5 vol 10% (w/v) ammonium acetate in methanol at −20°C for 3 h, and then centrifuged at 12,000 × g for 15 min at 4°C. The pellet was rinsed 3 times with acetone and then solubilized in lysis buffer [9 M urea, 2 M thiourea, 2% (w/v) CHAPS, 1% (w/v) DTT and 1% (v/v) carrier ampholytes of pH 3-10 (Bio-Rad, USA)]. The protein concentration was determined according to Bradford's method using BSA as standard (Bradford, 1976).

2-DE was performed with 800 μg protein samples. The proteins were mixed with 350 μL rehydration buffer [7 M urea, 4% (w/v) CHAPS, 2% (w/v) DTT, 2% (v/v) IPG buffer (pH 3-10), 0.001% (w/v) bromophenol blue], and then rehydrated overnight with the IPG strips (BioRad, 17 cm, pH 3-10 linear; Chan et al., 2007; Wang et al., 2014). Electrofocusing was carried out at 250 V for 30 min, followed by 1 kV for 1 h and 10 kV for 6 h using the PROTEAN IEF CELL system (Bio-Rad, USA) at 20°C. Prior to the second dimensional electrophoresis, the IPG strips were equilibrated with two equilibration buffers [50 mM Tris-HCl (pH 8.8), 7 M urea, 10% (w/v) glycerol and 2% (w/v) SDS] for 15 min. Buffer 1 contained 2% (w/v) DTT, whereas buffer 2 contained 2.5% (w/v) iodoacetamide. Then the strips were loaded and run on 12% separation gels and 5% stacking gels at a constant voltage of 150 V using the PROTEAN II Xi Cell system (Bio-Rad, USA). The gel was stained overnight with 1% (w/v) G-250 Colloidal Coomassie Blue solution containing 50% (v/v) ethanol and 15% (v/v) acetic acid (Li et al., 2015). At least three biological replicates were used for each treatment and at least one gel was run for each replicate.

### Gel scanning and image analysis

The stained gels were scanned using UMAX 2100XL-USB scanner (Bio-Rad, USA) at a resolution of 600 dpi. The gel images were saved in TIF format, and analyzed using PDQuest 2-D analysis software (Version 8.0, Bio-Rad, USA). Three gels with well-separated protein spots of each treatment were used to create “replicate groups” (Chandramouli et al., 2011; Wang et al., 2014). To obtain the highest gel matching, manual editing was carried out after automated detection and matching. Control was used as reference gel. To account for quantitative variations, the data were normalized between samples using total intensity of protein spots based on the corresponding gel (Chandramouli et al., 2011). The normalized intensity of protein spots was averaged from 2-DE gels for three independent biological replicates. For statistical analysis, the SPSS software 22.0 (IBM SPSS, USA) was employed. Spots differing by 2-fold or greater volume between treatments were excised and used for protein identification (Li et al., 2015).

### Mass spectrometry (MS) analysis and database searching

Spots which showed quantitative statistically significant differences between treatments were subjected to MS analysis (4700 proteomics Analyzer, Applied Biosystems, USA). The differently accumulated spots were manually excised from the 2-D gels, and then digested with trypsin (Promega, USA) (Wang et al., 2014; Li et al., 2015). MS spectra were acquired using the positive ion reflector mode with resolution ratio at 50,000 over the full-scan spectrum. The five most abundant precursor ions were selected for MS/MS scans. All acquired spectra were processed using Flex Analysis 3.3 software (Bruker) (Romero-Rodríguez et al., 2015). Database searching, including PMF and MS/MS, were performed using Mascot software 2.3.02 (Matrix Science, UK) against Cucumber Genomic database with 66,197 sequences and 26033094 residues (http://cucurbitgenomics.org/). The database parameters for searching were as follows: Trypsin, Carbamidomethyl (C) as a fixed modification and Oxidation (M) as a variable modification, 100 ppm mass tolerance in MS and 0.7 Da for MS/MS data, 1 max missed cleavage. The cut-off score for accepting individual MS/MS spectra: >61 (*p* < 0.05) (Jamesdaniel et al., 2012). Results with the highest score were considered as relevant for each identified protein. All the peptide matches for each spot were provided in Table S2. The mass spectrometry proteomics data have been deposited to the ProteomeXchange Consortium via the PRIDE partner repository with the dataset identifier PXD008551 (Submission details - Project Name: Proteomic analysis of cucumber (*Cucumis sativus*) fruit peel in response to pre-storage cold acclimation, Project accession: PXD008551, Project DOI: Not applicable, Reviewer account details - Username: reviewer80830@ebi.ac.uk, Password: CcmdYGZm). Proteins identified by MALDI TOF/TOF analysis were classified based on their putative function using Blast2GO (Li et al., 2015). The mass (*Mr*) and isoelectric point (*pI*) were determined with the online analysis tool Expasy (http://web.expasy.org/compute_pi/).

### RNA extraction and real time-PCR analysis

Total RNA was extracted using Trizol reagent (Invitrogen, USA) according to the manufacturer's instructions (Xia et al., 2009). Total RNA was treated with DNaseI and reverse-transcribed using iScript cDNA Synthesiskit (Bio-Rad, USA). Quantitative real time-PCR analysis was carried out with the SYBR Green PCR Master Mix (Bio-Rad, USA) according to the method we described previously (Wang and Zhu, 2017). To measure reaction efficiency, the standards of 1,000, 100, 10, 1, and 0.1 ng of total starting RNA were used to generate a standard curve according to the method described by Wacker and Godard (2005). For each gene, a standard curve with log of the RNA concentration on the X-axis and cycle threshold on the Y-axis was generated. A line with best fit was done using five concentration data points. The slope of the line as well as R^2^ values were calculated using Bio-Rad CFX manager (version 2.1) program. Gene-specific primers are listed in Table S1. Expression values were normalized using *Actin*.

### Statistical analysis

Experiments were conducted with three independent biological replicates. Statistical analysis was performed using SPSS software (Version 22.0). Least significant differences (LSDs) were calculated to compare significant effects. Differences at *P* < 0.05 were considered to be significant.

## Results

### Effects of PsCA treatment on chilling severity of cucumber fruit

**Figure 1:**
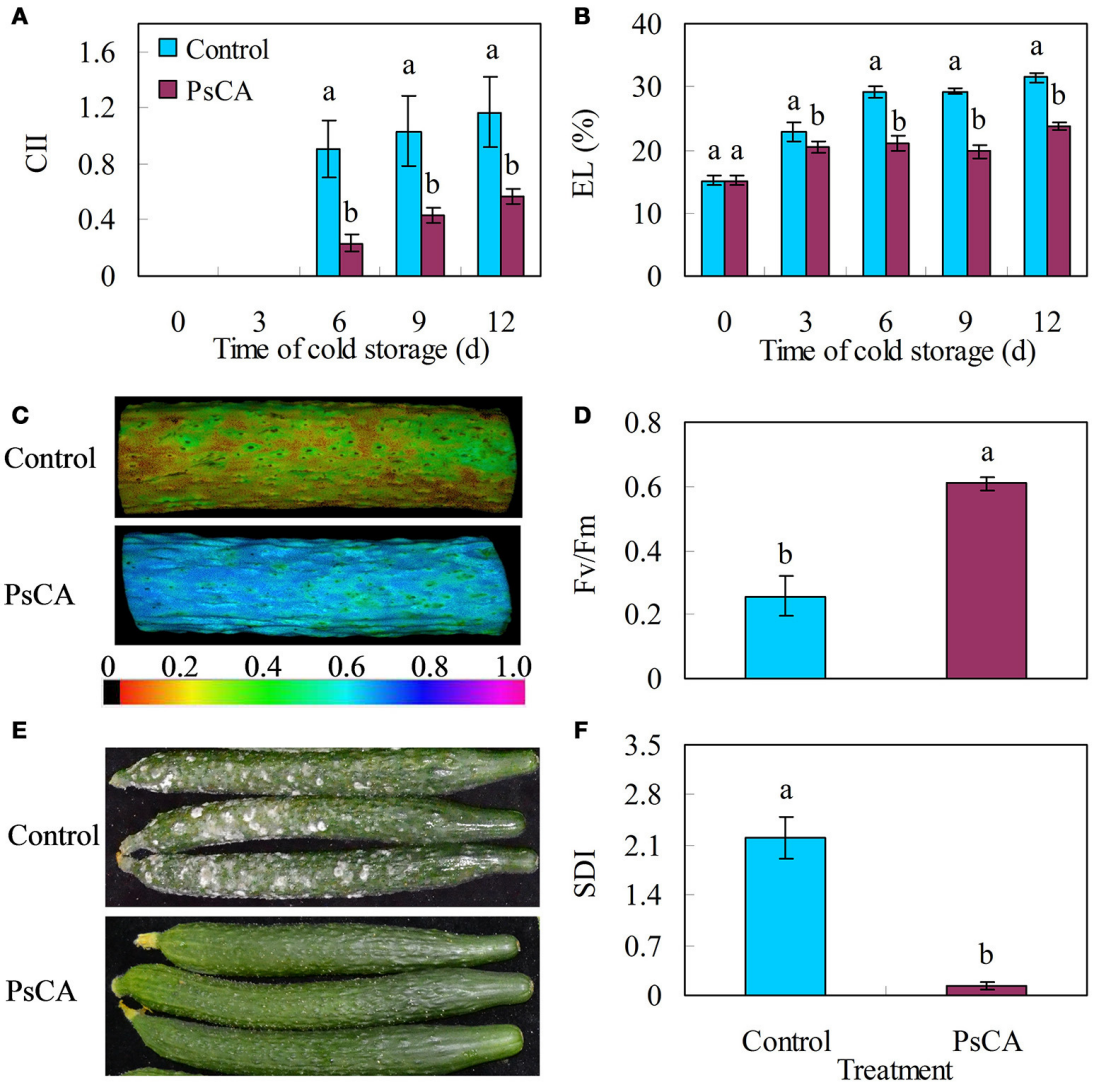
Chilling severity of harvested cucumber. **(A)** chilling injury indices (CII); **(B)** electrolyte leakage (EL); **(C,D)** images and values of the maximum PSII quantum yield (Fv/Fm), evaluated after 12 d storage at 5°C. The color code depicted at the bottom of the image ranged from 0 (left) to 1.0 (right); **(E,F)** secondary disease pictures were taken and severity evaluated on the 4th d after the cucumbers were transferred to ambient temperature (20°C) following 12 d of storage at 5°C. Control fruit were directly placed at 5°C. Pre-storage cold acclimation (PsCA) treated fruit were first incubated at 10°C for 3 d and then stored at 5°C. Significance of differences between the control and PsCA are indicated by letters above the bars (*P* ≤ 0.05). Data are presented as means ± standard errors (*n* = 3).

Chilling injury develops when cucumber fruit are stored at temperatures below 7–10°C (Yang et al., 2011). Chilling injury leads to loss of cell integrity of the peel, causing electrolyte leakage (EL), which can be used as a quantitative indicator for chilling injury (Cao et al., 2012; Liu et al., 2016). Surface pitting is the only chilling injury symptom of cucumber fruit and is not very obvious under low temperature (Wang and Zhu, 2017). However, the injury will be aggravated and lead to decay when the fruit are transferred to ambient temperature from low temperature (DeEll et al., 2000). Therefore, the secondary disease index (SDI) is also used as an indicator for chilling injury (Wang and Zhu, 2017). Chlorophyll fluorescence is an indirect measurement of the physiological status of chlorophyll-containing tissues. The maximal PSII quantum yield (Fv/Fm) is a chlorophyll fluorescence parameter. This ratio reflects the maximum potential quantum efficiency of Photosystem II and a decline in Fv/Fm is related a loss of photosynthetic activity in fruit during cold storage (Huang et al., 2012; Li et al., 2015), and therefore, Fv/Fm has been widely used for evaluating cold tolerance in plants (Xia et al., 2009; Li et al., 2015; Wang et al., 2016). Compared with the control, PsCA treatment reduced CII and electrolyte leakage by 51.43 and 24.55% respectively at the end of cold storage (Figures 1A,B). Meanwhile, PsCA-treated cucumber had an increased ratio of Fv/Fm compared to the control (Figures 1C,D). Moreover, the secondary disease severity (SDI) of PsCA-treated cucumbers was nearly 94% lower than that of the control 4 d after being transferred to 20°C following cold storage at 5°C (Figures 1E,F).

### Differentially accumulated proteins induced by PsCA

**Figure 2:**
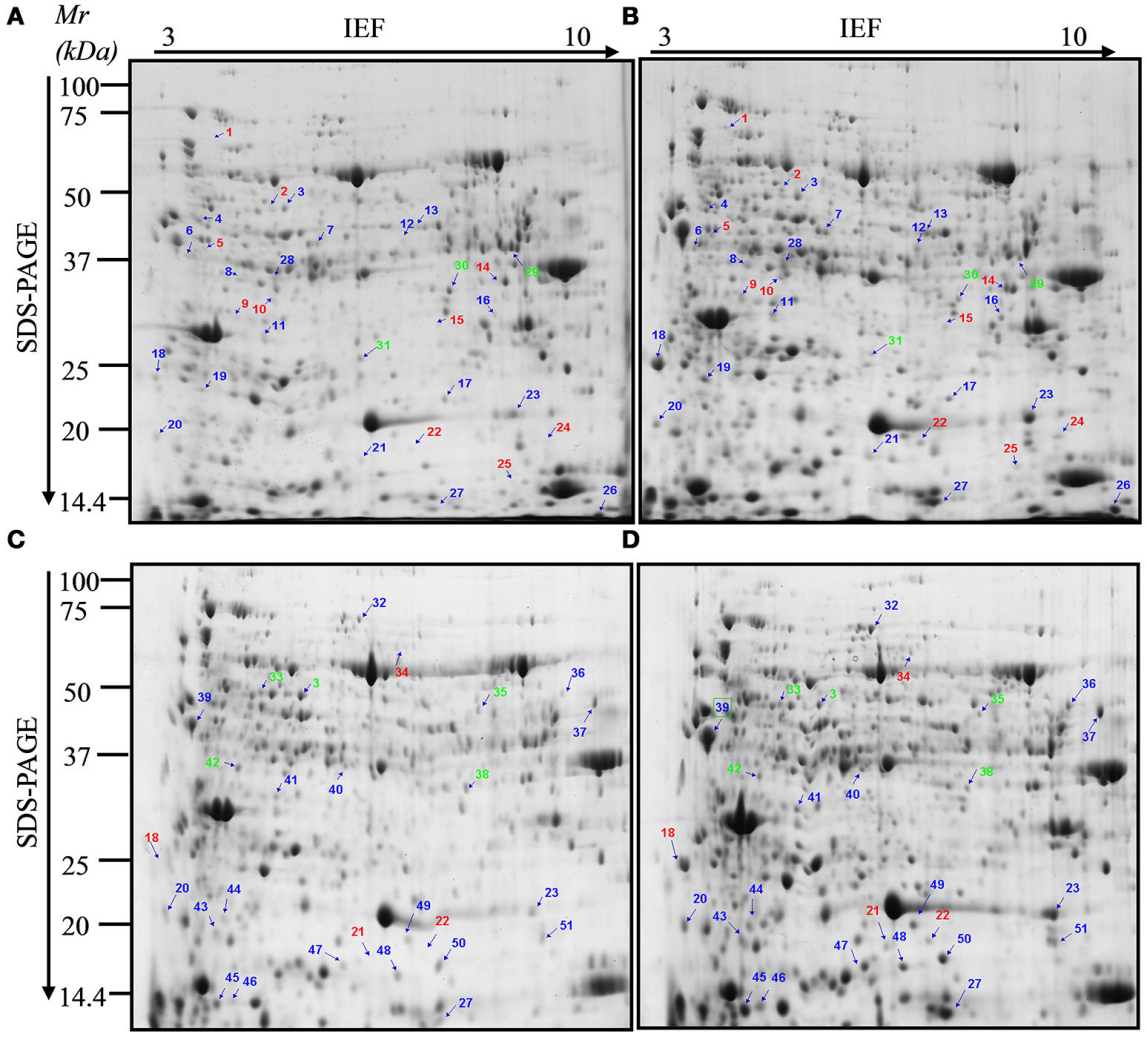
Two-dimensional electrophoresis maps of the differentially accumulated proteins in harvested cucumber fruit. **(A)** Total protein from the non-acclimated fruit (NA); **(B)** Total protein from fruit treated with 3 d of PsCA; **(C,D)** Total protein from the control (12 d in cold storage) and the fruit exposed to PsCA treatment plus 9 d of storage at 5°C, respectively. Non-acclimation (NA) samples show the proteomic profile prior to PsCA treatment. Control fruit were directly placed at 5°C. Individual spots are indicated by arrows and numbers. These spot numbers correspond to those in Tables 1, 2. Blue numbers indicate that the corresponding proteins were up-regulated after PsCA treatment, while green numbers indicate down-regulation. Red numbers indicate proteins that were only detected in PsCA-treated fruit.

In order to distinguish PsCA-responsive proteins, total proteins extracted from the non-acclimated (before PsCA treatment, hereafter referred to as NA) fruit, the fruit exposed to 3 d of cold acclimation (hereafter referred to as PsCA), the control fruit stored at 5°C for 12 d (Control) and the fruit exposed to 3 d of PsCA treatment followed by 9 d of cold storage (or PsCA-treated and cold stored fruit, hereafter referred as PsCA+CS) were separated by 2-DE, respectively. 2-DE gels of three independent biological replicates were obtained and analyzed (Figure S1). More than 750 protein spots were detected using PDQuest Advanced 2-D Gel Analysis software (Version 8.0) following the 2-DE gel analysis (Figure 2 and Figure S1). Of the all detected spots, 58 that displayed significant and reproducible changes in abundance (see the numbered protein spots in Figures 2A–D) were isolated, and 56 spots were successfully characterized using MS/MS coupled with database searching, which matched 51 proteins. 38 proteins have scores greater than threshold scores of 61 (*P* < 0.05; Tables 1, 2) and they are regarded as the significant differentially—accumulated proteins (SDAPs). Furthermore, in order to ensure the reproducibility of the results, for a set of protein spots (spots 3, 18, 20, 21, 22, 23, and 27), regions of gel from PsCA+CS fruit (Table 2 and Figure 2) that corresponded to same regions of gel from PsCA fruit (Table 1 and Figure 2) were excised, and proteins extracted and analyzed by MS. The results show that specific spots from two different gels match the same proteins (Tables 1, 2).

**Table 1:** Identification of the differentially accumulated proteins by MS from the peel from harvested cucumber fruit exposed to 3 d of PsCA treatment in comparison to non-acclimated fruit (NA, 0 h of PsCA treatment).

**Spot no**.	**Fold change^a^**	**Protein description**	**NCBI accession**	**Mr(mass)/pI^b^**	**Score^c^**	**NP^d^**
18(↑)	21.8	Chitinase	gi|167539	30.77/4.46	89^*^	6
19(↑)	8.47	Hypothetical protein Csa_3G824890	gi|700204411	36.7/4.68	158^*^	11
16(↑)	4.39	Glycine-rich RNA-binding protein 3	gi|449439327	18.24/9.54	59	5
27(↑)	3.32	Abscisic stress-ripening protein 1-like	gi|449441222	13.28/6.25	250^*^	7
20(↑)	3.13	Probable calcium-binding protein CML27	gi|449439129	20.57/4.33	167^*^	8
12(↑)	3.01	Fructose-bisphosphate aldolase	gi|778684132	40.16/8.1	210^*^	7
21(↑)	2.95	Serine–tRNA ligase	gi|778697947	51.53/6.33	138^*^	6
8(↑)	2.78	Enoyl-[acyl-carrier-protein] reductase [NADH]	gi|449448774	41.41/8.64	50	3
7(↑)	2.69	Glutamine synthetase	gi|374676432	39.28/5.82	108^*^	4
13(↑)	2.56	Formate dehydrogenase	gi|700204533	42.2/6.71	110^*^	5
11(↑)	2.42	Cysteine protease	gi|700206717	40.73/6.07	104^*^	3
17(↑)	2.30	Superoxide dismutase [Mn]	gi|700208822	26.88/7.88	110^*^	5
23(↑)	2.16	Thaumatin-like protein	gi|700194957	24.23/7.35	238^*^	6
26(↑)	2.10	Oxygen-evolving enhancer protein 3-2	gi|449454700	24,71/9.42	203^*^	5
28(↑)	2.06	Peroxidase 2	gi|129810	31.86/6.09	63^*^	3
4(↑)	1.99	DNA damage-inducible protein 1 isoform	gi|778725514	44.69/4.92	150^*^	6
3(↑)	1.89	*S*-adenosylmethionine synthetase	gi|778728392	43.22/5.35	196^*^	6
1	DOIP	Hypothetical protein Csa_3G681140	gi|700203416	50.69/6.72	33	3
2	DOIP	Protein transport protein SEC31 homolog B	gi|449464024	120.58/5.16	37	4
5	DOIP	Sedoheptulose-1,7-bisphosphatase	gi|229597543	42.1/5.96	68^*^	3
6	DOIP	Hypothetical protein Csa_1G586820	gi|700211124	53.17/9.46	42	5
9	DOIP	Uncharacterized protein LOC101209635 (universal stress protein family)	gi|449458209	27.71/5.31	66^*^	1
10	DOIP	Hypothetical protein Csa_1G662500	gi|700211614	7.04/9.59	24	1
14	DOIP	Polyprotein (coat protein)	gi|8439505	41.36/6.62	114^*^	6
15	DOIP	Xyloglucan endotransglycosylase	gi|480296299	32.64/6.42	48	3
22	DOIP	Non-identified				
24	DOIP	Glutathione peroxidase	gi|206604173	20.66/8.9	42	1
25	DOIP	Glycine-rich RNA-binding protein	gi|700208956	14.76/9.95	30	2
29(↓)	0.32	Glyceraldehyde-3-phosphate dehydrogenase	gi|449441394	36.68/7.02	130^*^	5
30(↓)	0.35	Annexin A6	gi|449449278	36.36/6.61	152^*^	10
31(↓)	0.23	Gamma carbonic anhydrase 1	gi|449469963	29.74/5.99	220^*^	10

a *Average fold change of a protein abundance between 3 d of PsCA treatment vs. non-acclimation (NA). DOIP: corresponding spots detected only in the PsCA-treated fruit*.

b *The mass (kDa) and pI of identified proteins*.

c Protein scores/Expectation reported after searching against the Cucumber Genomic database. Protein scores >61 were marked with ^*^.

d *The number of matched peptides*.

**Table 2:** Identification of the differentially accumulated proteins by MS in peel from harvested cucumber fruit exposed 3 d of PsCA treatment plus 9 d of cold storage at 5°C in comparison to the control that were directly stored at 5°C for 12 d.

**Spot no**.	**Fold change^a^**	**Protein description**	**NCBI accession**	**Mr(mass)/pI^b^**	**Score^c^**	**NP^d^**
18(↑)	32.09	Chitinase	gi|167539	30.77/4.46	89^*^	6
23(↑)	8.10	Thaumatin-like protein	gi|700194957	24.23/7.35	238^*^	6
27(↑)	6.14	Abscisic stress-ripening protein 1-like	gi|449441222	13.28/6.25	250^*^	7
20(↑)	2.73	Probable calcium-binding protein CML27	gi|449439129	20.57/4.33	167^*^	8
21	DOIP	Serine–tRNA ligase	gi|778697947	51.53/6.33	138^*^	6
22	DOIP	Non-identified				
3(↓)	0.27	*S*-adenosylmethionine synthetase	gi|778728392	43.22/5.35	196^*^	6
47(↑)	6.69	Peroxiredoxin-2B-like	gi|449449525	17.29/5.77	147^*^	7
36(↑)	5.37	Cyclin-dependent kinase 13-like	gi|449466564	38.74/7.77	51	1
44(↑)	4.28	Thylakoid lumenal protein At1g12250	gi|449459702	29.92/8.59	207^*^	11
49(↑)	3.57	Glycine-rich protein 2	gi|778722923	19.68/6.29	264^*^	5
34(↑)	3.15	Trithorax group protein osa	gi|449438092	56.31/6.16	69^*^	5
48(↑)	3.15	Transcription factor BTF3 homolog 4-like isoform X2	gi|449439239	17.46/6.62	96^*^	6
43(↑)	2.93	Non-identified				
45(↑)	2.90	Nodulin-related protein 1	gi|700196786	14.96/5.21	223^*^	8
51(↑)	2.78	Glutathione peroxidase	gi|206604173	20.66/8.9	134^*^	2
50(↑)	2.47	Transcription factor BTF3 homolog 4-like	gi|449439495	17.4/6.74	95^*^	5
40(↑)	2.46	Oxygen-dependent coproporphyrinogen-III oxidase	gi|449459988	44.97/6.65	263^*^	9
41(↑)	2.42	Probable L-ascorbate peroxidase 6	gi|778715658	44.77/7.09	227^*^	10
39(↑)	2.33	Peroxidase	gi|700198939	34.3/4.94	338^*^	7
32(↑)	2.31	Hsp70-Hsp90 organizing protein 3-like	gi|449460409	64.94/5.78	260^*^	5
46(↑)	2.27	Superoxide dismutase [Cu-Zn]	gi|700191683	22.62/5.87	209^*^	4
37	DOIP	Extra-large guanine nucleotide-binding protein 1-like isoform X3	gi|778669208	79.90/5.47	43	5
33(↓)	0.37	S-adenosylmethionine synthetase 2	gi|449472806	43.22/5.35	94^*^	6
35(↓)	0.33	Obg-like ATPase 1	gi|449435526	44.41/6.25	252^*^	9
38(↓)	0.31	Annexin A6	gi|449449278	71.97/6.61	154^*^	10
42(↓)	0.38	Lactoylglutathione lyase	gi|778720394	32.66/5.23	125^*^	4

a *Average fold change of a protein abundance between 3 d of PsCA treatment vs. non-acclimation (NA). DOIP: corresponding spots detected only in the PsCA-treated fruit.*.

b *The mass (kDa) and pI of identified proteins*.

c Protein scores/Expectation reported after searching against the Cucumber Genomic database. Protein scores >61 were marked with ^*^.

d *The number of matched peptides*.

### Proteomic changes caused by PsCA treatment relative to non acclimation

**Figure 3:**
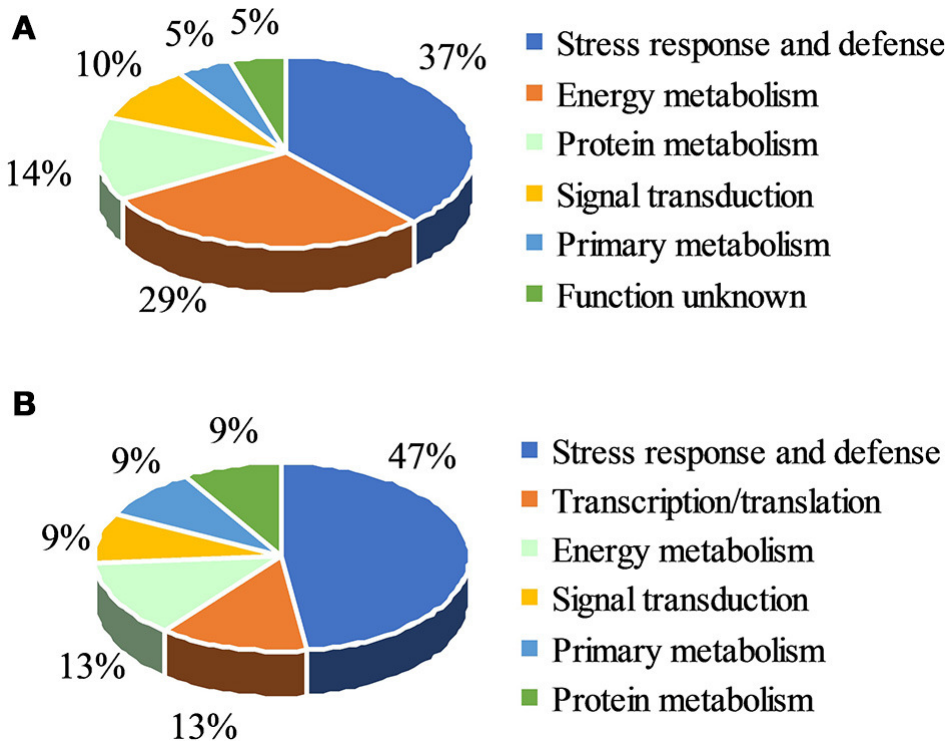
Functional classification of differentially accumulated proteins. **(A)** Classes of significant differentially accumulated proteins (SDAP) in Table 1; **(B)** Classes of SDAP s in Table 2. The identified proteins were classified using Blast2Go.

Among the total of 31 protein spots that showed significant volume changes, 21 were SDAPs from comparison between PsCA fruit and NA fruit (Compare Figure 2A with Figure 2B). Of the 21, 15 spots were up-regulated, three spots down-regulated, and three spots visible only in PsCA fruit (Figures 2A,B, Table 1). To generate an overview of the most relevant biological processes related to PsCA, a categorization of these SDAPs was performed. Twenty of the SDAPs were already known proteins and functionally characterized, while one [polyprotein (coat protein) (spot 14)] was not characterized as with known function in plants. The 20 known SDAPs were related to stress response and defense (SRD) (37%), energy metabolism (29%), protein metabolism (14%), signal transduction (10%), primary metabolism (5%) (Figure 3A).

The eight stress- and defense-associated proteins were all up-regulated or newly synthesized. The seven upregulated proteins were DNA damage-inducible (DDI) protein 1 (spot 4), superoxide dismutase [Mn] (spot 17), chitinase (spot 18), Hypothetical protein Csa_3G824890 (spot 19), thaumatin-like protein (spot 23), abscisic stress-ripening protein 1-like (spot 27), peroxidase 2 (spot 28), and one de novo synthesized was uncharacterized protein LOC101209635 (universal stress protein family). Of the six SDAPs involved in energy metabolism, four [sedoheptulose-1,7-bisphosphatase (spot 5), fructose-bisphosphate aldolase (spot 12), formate dehydrogenase (spot 13), oxygen-evolving enhancer protein 3-2 (spot 26))] were up-regulated, while two [glyceraldehyde-3-phosphate dehydrogenase (spot 29), gamma carbonic anhydrase 1, mitochondrial (spot 31)] were down-regulated. The three SDAPs involved in protein metabolism [glutamine synthetase (spot 7), cysteine protease (spot 11), serine–tRNA ligase (spot 21)] were all up-regulated. The one SDAP involved in primary metabolism, *S*-adenosylmethionine synthetase 2 (spot 3), was up-regulated. As for the two SDAPs related to signal transduction, probable calcium-binding protein CML27 (spot 20) was up-regulated and annexin A6 (spot 30) was down-regulated.

### Proteomic differences between the control and acclimated cucumber following cold storage

Following cold storage, a total of 27 protein spots showed significant changes in abundance, of which 23 had scores >61. These SDAPs fall into six classes, i.e., stress response and defense (SRD) (47%), transcription (13%), energy metabolism (13%), signal transduction (9%), primary metabolism (9%), and protein metabolism (9%) (Figure 3B).

Among the 11 SDAPs involved in SRD, 10 were up-regulated and one down-regulated. The 10 upregulated proteins were thaumatin-like protein (spot 23), abscisic stress-ripening protein 1-like (spot 27), chitinase (spot 18), peroxiredoxin-2B-like (spot 47), nodulin-related protein 1 (spot 45), glutathione peroxidase (spot 51), probable L-ascorbate peroxidase 6 (spot 41), peroxidase (spot 39), superoxide dismutase [Cu-Zn] (spot 46), and glycine-rich protein 2 (spot 49). The one down-regulated protein was lactoylglutathione lyase (spot 42). The three transcription-associated proteins [trithorax group protein osa (spot 34), transcription factor BTF3 homolog 4-like isoform X2 (spot 48), and transcription factor BTF3 homolog 4-like (spot 50)] were all up-regulated. Three SDAPs were involved in energy metabolism, of which two [thylakoid lumenal protein At1g12250 (spot 44) and oxygen-dependent coproporphyrinogen-III oxidase, chloroplastic (spot 40)] were up-regulated, and one [obg-like ATPase 1 (spot 35)] was down-regulated. Of the two signal transduction-related proteins, probable calcium-binding protein CML27 (spot 20) was up-regulated, while annexin A6 (spot 38) was down-regulated. The two *S*-adenosylmethionine synthetases (spot 3 and spot 33) involved in primary metabolism methionine cycle were both down-regulated. Of the two SDAPs involved in protein metabolic process, hsp70-Hsp90 organizing protein 3-like (spot 32) was upregulated, and serine–tRNA ligase (spot 21) was only detected in PsCA-CS fruit.

### Effects of PsCA treatment on protein and transcript accumulation patterns of representative differentially accumulated proteins

**Figure 4:**
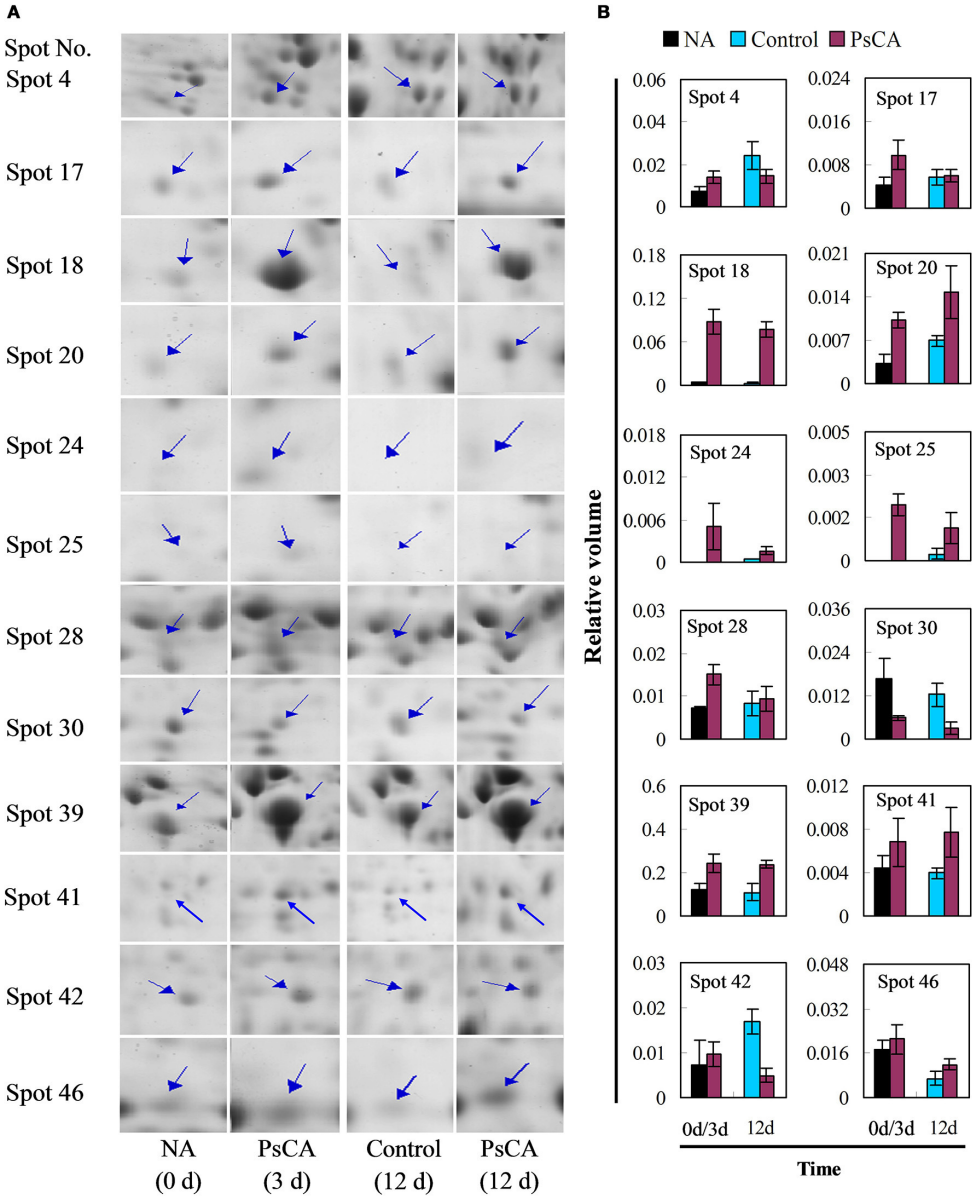
Enlarged views **(A)** and abundance quantifications **(B)** of representative differentially accumulated proteins marked in Figure 2. Non-acclimation (NA) was 0 h of PsCA treatment. Control fruit were directly placed at 5°C. PsCA treatment was a 3 d incubation at 10°C followed by storage at 5°C. The values of protein abundance were measured using PDQuest 8.0 software (Bio-Rad, USA), and were analyzed by SPSS (Version 22.0) (see Table S3). The blue arrows indicate the hypothetical position of protein spots. Their sample numbers followed the manuscript annotation. Data are presented as means ± standard errors (*n* = 3).

**Figure 5:**
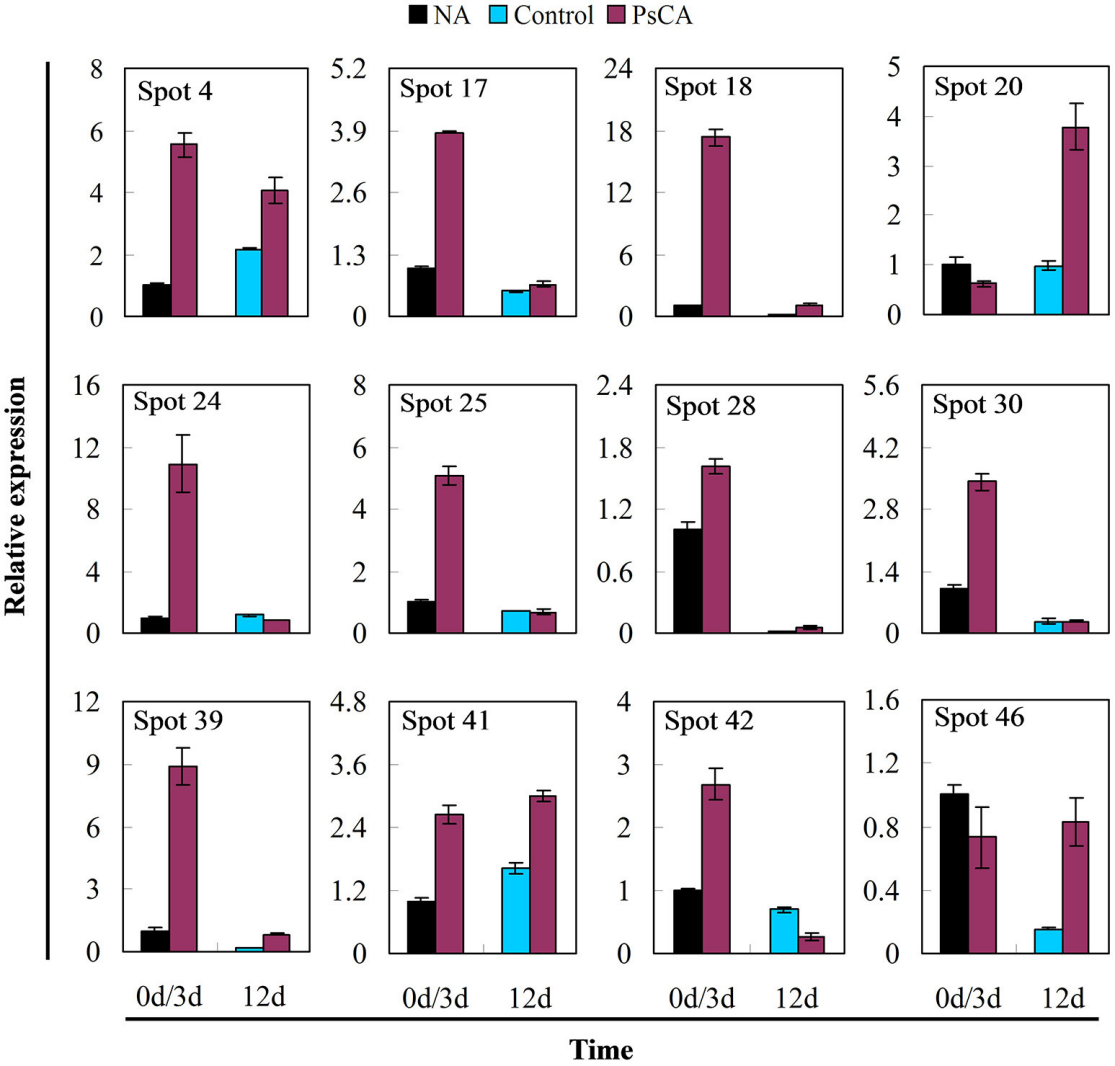
Effects of pre-storage cold acclimation (PsCA) on transcript accumulation of representative differentially accumulated proteins in cucumber. The relative transcript accumulation was evaluated by quantitative real-time PCR using gene-specific primers (see Table S1). Control fruit were directly placed at 5°C. PsCA treatment was first incubated at 10°C for 3 d and then stored at 5°C. The transcript accumulation data were all normalized to 100% (1.0) at 0 d (before cold treatment). Data are presented as means ± standard errors (*n* = 3).

Twelve differentially accumulated proteins were selected for investigation of transcript accumulation patterns using quantitative real-time PCR (**Figure 5** and Table S4). Of which, 10 proteins have scores >61 (SDAPs), while two have scores <61 (spots 24, 25). Among the eight proteins (spots 4, 17, 18, 20, 24, 25, 28, 39) significantly up-regulated after PsCA treatment (Figure 4), seven had increased levels of transcripts (Figure 5). Of the seven proteins (spot 18, 20, 24, 25, 39, 41, 46) that were significantly up-regulated after cold storage (Figure 4), five also had increased transcript levels (Figure 5). On the other hand, for the three proteins (spots 41, 42, 46) that did not show significant up-regulation after PsCA treatment, two (spots 41, 42) showed increases in their transcripts. For the two proteins (spots 17, 28) that were not significantly up-regulated after cold storage, neither showed significant changes in transcript levels after cold storage. However, while the protein abundance (spot 30) was significantly decreased after PsCA treatment, the corresponding transcript was up-regulated. Among the three proteins (spots 4, 30, 42) whose abundance decreased after cold response, only one (spot 42) showed a decrease in its transcript. In addition, the time-dependent changes in transcripts for these proteins were also monitored, which showed that all 12 proteins had corresponding increases in the transcript levels during cold storage (Figure S2 and Table S4).

## Discussion

In this study, PsCA treatment significantly reduced CII, EL, SDI, and Fv/Fm ratio in cucumber stored at chilling temperature (Figure 1). These results strongly suggest that cold acclimation induced chilling tolerance in cucumber.

Since it is the peel that is first exposed to temperature stress, proteomic profile changes in the peel were investigated to identify the responses of cucumber to cold acclimation. A total of more than 750 protein spots were detected, suggesting the proteins from cucumber peels were well separated. This is confirmed by the fact that about 97% of proteins spots (56 out of the 58) were identified by MS/MS analysis. Furthermore, when the corresponding regions to the seven protein spots (spots 3, 18, 20, 21, 22, 23, and 27) of PsCA treatment (Table 1) were retrieved from the gel from the PsCA+CS experiment, they matched the same proteins. These observations suggest that the 2-DE and the preparation of proteins were reproducible, providing a solid basis to justify the present results.

The 21 SDAPs identified as a result of 3 day of PsCA treatment and the 23 SDAPs identified from PsCA+CS vs. the control are classified into six categories, however, the largest category is related to SRD. Eight of the 21 SDAPs (37%) induced by PsCA treatment (Figure 3A), and 11 of the 23 SDAPs (47%) identified by PsCA+CS treatment (Figure 3B), were related to SRD. This suggests that the SRD category included the most proteomic profile changes brought about by PsCA. Furthermore, all but one SRD proteins were upregulated, suggesting accumulation of such proteins provide a key feature of PsCA-induced responses.

It is well established that chilling temperature leads to the accumulation of reactive oxygen species (ROS), which cause oxidative stress to damage cell structures (Ashraf, 2009; Valenzuela et al., 2017). Plants have developed a complex antioxidant system to cope with oxidative stress, which includes antioxidant enzymes such as superoxide dismutase (SOD), catalase (CAT), peroxidase (POD) as well as those involved in the ascorbate-glutathione cycle such as ascorbate peroxidase (APX), glutathione peroxidase (GPX), glutathione-S-transferase, gluthatione reductase, and also non-enzymatic antioxidants (Mittler, 2002; Ashraf, 2009; Valenzuela et al., 2017). SOD is one of the most important antioxidant enzymes used in plants against oxidative stress and catalyses the dismutation of the highly toxic superoxide radical to less toxic hydrogen peroxide (H_2_O_2_) and molecular oxygen (Alscher et al., 2002). CAT and POD are the key enzymes for scavenging H_2_O_2_ in plants (Mittler, 2002; Ashraf, 2009). In addition, peroxiredoxin-2B-like plays an important role in detoxification of H_2_O_2_ in *Arabidopsis* as a member of the peroxidase superfamily (Bréhélin et al., 2003). The enzymes of the ascorbate-glutathione cycle are also important to counteract oxidative stress in plants. APX is another key enzyme for scavenging H_2_O_2_ in plant cell (Narendra et al., 2006). GPX is also a potential scavenger of lipid radicals and ROS (Miao et al., 2006). In this study, SOD (spot 17), POD (spot 28) were significantly up-regulated and GPX (spot 7) newly synthesized in PsCA treatment (Figures 2A,B, Table 1). In addition, following 12 d of cold storage, PsCA+CS fruit peel showed higher accumulation of SOD (spot 46), POD (spot 39), peroxiredoxin-2B-like (spot 47), GPX (spot 51), and L-ascorbate peroxidase 6 (spot 41) than the control (Figures 1C,D, Table 2). These results collectively suggest that PsCA treatment activated ROS-scavenging system, which includes both antioxidant enzymes and the ascorbate-glutathione cycle, and thereby protected the cool-stored cucumber from oxidative stress, consistent with our previous studies using physiological and molecular approaches that showed that PsCA inhibits generation of ROS by enhancing enzymatic activities of SOD, POD, and APX (Wang and Zhu, 2017). Furthermore, the present study suggests that the up-regulated SOD, POD, and GPX play major roles in protecting cucumber from ROS stress during cold storage. It is noted that for some enzymes, such as SOD, POD, and GPX, the specific proteins identified following PsCA treatment differed from those after the following cold storage. This could be due to the existence of multiple forms of the same enzymes or isoenzymes.

Pathogenesis- related (PR) proteins are expressed in plants in response to biotic and abiotic stresses (Renaut et al., 2006) and several groups of PR proteins are responsive to low temperature. The PR protein 1b1 is a major protein responsive to chilling temperature in tomato fruit and is up-regulated in high polyamine transgenic genotypes (Goyal et al., 2016). Thaumatin-like proteins might play important roles against chilling stress in peach fruit (Dagar et al., 2010). Ethylene pretreatment significantly induced the expression of three thaumatin-like proteins in cool-stored banana fruit (Li et al., 2015). Several chitinases were accumulated during cold acclimation in winter rye (Yeh et al., 2000; Stressmann et al., 2004). In the current study, three PR proteins, namely chitinase (spot 18) and thaumatin-like protein (spot 23) were significantly up-regulated following PsCA treatment. As chitinase is a marker for systemic acquired resistance (SAR) (Busam et al., 1997; Carella et al., 2016), this implies that exposure to PsCA induced SAR in cucumber, which contributed to the much reduced severity of secondary diseases following transfer of cold-stored cucumbers into ambient temperature. The increased abundances of chitinase (spot 18) and thaumatin-like protein (spot 23) in PsCA+CS cucumber than in the control following cold storage imply that these two PR proteins play an important role in defense against fungal infection. In addition, a nodulin-related protein 1 (NPR1, spot 45) was more abundant in PsCA+CS fruit peel than in the control. As NRP1 was required for defense responses against cold stress and avirulent bacteria (Quirino et al., 2004; Fu et al., 2010), this suggests that NRP1 may play a role in PsCA-induced defense against chilling and bacterial diseases.

The universal stress protein (USP) domain is a superfamily of conserved genes which can be found in bacteria, protozoa, archaea, fungiand plants (Nachin et al., 2005). USP proteins are induced by many environmental stresses such as drought, extreme temperatures, salt and UV light (Nachin et al., 2005). The USPs enhance cell survival rate during exposure to such conditions as they can alter the expression of a variety of genes that help to cope with stress (Tkaczuk et al., 2013). In this study, a uncharacterized protein LOC101209635 (spot 9) containing one USP conserved domain was newly synthesized following 3 d of PsCA treatment, suggesting that PsCA enhanced tolerance to drought, extreme temperatures, salt or UV stresses.

Abscisic stress ripening protein 1 (ASR1) is a low molecular weight plant-specific protein whose expression is regulated by abiotic stress (Goldgur et al., 2007). Most ASRs and orthologs are regulated by ABA (Dóczi et al., 2005). Studies have shown that cold acclimation induces chilling tolerance mainly through an ABA-independent pathway (Thomashow, 2010). However, cold acclimation induces ABA accumulation in *Arabidopsis* (Cuevas et al., 2008). Overexpression of *ASR1* in transgenic plants increases their salt and water tolerance (Goldgur et al., 2007). Hypothetical protein Csa_3G824890 shares 47% sequence similarities with uncharacterized protein At5g39570 (NCBI No.: XP_020872957.1). Recently, the protein At5g39570 has been characterized as phospholipase D-regulated protein1 (PLDrp1) in *Arabidopsis*, which showed a high content of glycine (17.7%) at the N-terminal region and transcripts were accumulated in response to water stress (Ufer et al., 2017). In the present study, an abscisic stress-ripening protein 1-like (spot 27) was up-regulated following 3 d of PsCA treatment and was much more abundant in PsCA+CS cucumber than in the control, implying PsCA could induce tolerance to abiotic stresses, such as chilling, salt, and water stresses.

In plants, glycine-rich proteins (GRPs) are implicated in plant defense and modulated by biotic and abiotic factors (Sachettomartins et al., 2000). GRPs are proposed to be a part of the defense and repair system of plants (Mousavi and Hotta, 2005). *Craterostigma plantagineum* glycine-rich protein 1 accumulates in response to dehydration and is essential for the acquisition of desiccation tolerance (Giarola et al., 2016). In the current study, glycine-rich protein 2 (spot 49) displayed higher intensity in PsCA+CS cucumber than in the control, suggesting PsCA induced tolerance to dehydration or activated repair system in cucumber. Since reducing water loss delayed chilling injury in grapefruit (Purvis, 1984), the upregulated GRP 2 could be connected to enhanced chilling tolerance. As GRPs are thought to be involved in protoxylem repair in plants (Ryser et al., 1997), implying cell wall repair may contribute to the enhanced chilling tolerance in harvested cucumber fruit.

DNA damage is normally detrimental to living organisms. The repair of DNA damage is essential for living organisms under biotic stress as well as abiotic stress. DDI proteins are DNA repair proteins, which can also regulate gene expression during plant defense responses (Ding et al., 2016). In the present study, a DDI protein 1 isoform (spot 4) was found to be up-regulated by PsCA, suggesting PsCA activated the DNA repair mechanism, which makes it possible to keep DNA intact and is especially important for cucumber fruit as a reproductive organ.

Lactoylglutathione lyase catalyzes the conversion of hemimercaptal, formed from methylglyoxal and glutathione, to S-lactoylglutathione (Dixon et al., 1998). It has been reported that methylglyoxal is a toxic compound under salt stress condition (Yadav et al., 2005). In the present study, PsCA+CS cucumber had less lactoylglutathione lyase (spot 42) than the control. This implies that in PsCA-treated cucumber less methylglyoxal is accumulated than in the control and there is less need for detoxification, which could well account for the reason why more glutathione, one of the most important non-enzymatic antioxidant in plants, is accumulated in PsCA-treated cucumber (Wang and Zhu, 2017). Therefore, the reduced level of lactoylglutathione lyase could be an indication of higher chilling tolerance.

From the above discussion, the eight SDAPs induced by 3 d of PsCA are not only related to defense against chilling stress, they are also involved in responses to salt and water stress, dehydration, fungi, bacteria, and insects, and in protecting DNA, suggesting the cucumber fruit is over-reacted to PsCA treatment. However, following cold storage, the 11 SDAPs involved in SRD were related only to defense against chilling and pathogens, suggesting that long-term cold storage caused the fruit to adapt itself to focus on the real stresses it actually faces.

Apart from the defense-related proteins, PsCA induced SDAPs involved in other processes, such as energy metabolism, protein metabolism, transcription regulation, signal transduction, and primary metabolism, suggesting PsCA induced defense responses involve coordination of complex physiological, biochemical, and molecular networks. After PsCA treatment, energy-metabolism-related SDAPs made up 29% of all SDAPs, while after cold storage, they made up only 13%, suggesting energy metabolism was very active during initiation of defense systems by PsCA treatment, but became much less active when the defense systems has already been established. After cold storage treatment, transcription-related proteins made up 13% of SDAPs, the second largest category, while right after PsCA, none of them have scores higher than the threshold of 61, suggesting that the PsCA treatment had a strong aftermath effect on transcription even after 9 d in cold storage. Proteins related to signal transduction made up almost the same percentage (9 or 10%) of SDAPs in PsCA-treated and PscA+CS fruit, suggesting that signal transduction is stably involved in PsCA induced defense response. As for primary metabolism, *S*-adenosylmethionine synthetase (SAM) is involved in PsCA-treated fruit and PsCA+CS fruit, suggesting that SAM plays an essential role in regulating PsCA-induced defense responses. SAM is an intermediate in the biosynthesis of polyamines and of the phytohormone ethylene (Ravanel et al., 1998). Polyamines are involved in plants' responses to abiotic stresses such as salinity (Tanou et al., 2014) and cold (Guo et al., 2014). Ethylene plays important roles in various abiotic stress responses in plants (Shi et al., 2012). Therefore, the present study implies that PsCA enhanced chilling tolerance through regulating biosynthesis of polyamines and ethylene.

In this study, 12 differentially accumulated proteins were selected for monitoring the changes in transcript level and seven of the significantly up-regulated proteins in PsCA treated fruit also accumulated transcripts (five proteins for PsCA+CS fruit). However, for the proteins that were down-regulated or not significantly up-regulated after PsCA or cold storage, the trend in transcript accumulation was different from that of the proteins. Overall, for the majority of the up-regulated differentially accumulated proteins, the trends at protein level were highly consistent with those at transcript level.

In conclusion, application of postharvest cold acclimation to cucumber induced significant chilling tolerance in cucumber. Proteomic analysis showed that cold acclimation induced at least six categories of significant differentially-accumulated proteins, and proteins related to SRD made up the biggest percentage of all categories, suggesting stress, and defense response makes up the largest component of the proteomic profile changes. In fruit just acclimated, the SRD proteins were related to responses to chilling, salt and water stress, dehydration, fungi, bacteria and insect, and protection of DNA. However, after cold storage, the SRD proteins in acclimated fruit were narrowed down in scope to those involved in defense against chilling and pathogens.

## Author contributions

SZ: conceived and oversaw the work; BW and FS: performed the experiments; BW: made the tables and figures; SZ and BW: wrote the manuscript. All authors have read and approved the manuscript.

### Conflict of interest statement

The authors declare that the research was conducted in the absence of any commercial or financial relationships that could be construed as a potential conflict of interest.
