# Supplementary material for: Proteomic Analysis of Differentially Accumulated Proteins in Cucumber (Cucumis sativus) Fruit Peel in Response to Pre-storage Cold Acclimation
Source: Front Plant Sci. 2018 Jan 18;8:2167. doi: 10.3389/fpls.2017.02167 (PMC5778441; doi:10.3389/fpls.2017.02167)
Supplement: Supplementary file 1 [file Table1.doc]

**Supplementary material**

**Table S1 |** Primer sequences used in quantitative real-time PCR.

| Spot no. | Forward primer(5＇–3＇) | Reverse primer (5＇–3＇) |
| --- | --- | --- |
| 4 | TCACTGTGATGACAGCCGAC | AGCAGAGCCTTCACGTTCTC |
| 16 | GCAGCCTTCCAAGCTGTCTA | ACTTGCTGAACGCTACCCTC |
| 17 | TTACAGGGCTCTGGATGGGT | CCAAGCAGAGGCACTAAAGC |
| 18 | AGCTCTTTAAGCAATGGCTGC | ATAGATGGCGATTCCAGCCG |
| 20 | CAACCCGATTCCCACCTCAA | AACCTTGCGGAGTTCTTCGT |
| 24 | CAAGGGCCACGGATTTGAGA | CTTGAAACGGGTGCAAGCAA |
| 25 | GCAGCCTTCCAAGCTGTCTA | ACTTGCTGAACGCTACCCTC |
| 28 | AATCATGCCCCGACGTATCC | AAGCTCACTCACCACACCAG |
| 30 | ATTCCGCACCTTAATCGGCA | TTTCTGTAACCCAGGACGGC |
| 39 | TCAGCGGACGCCTAAACAAT | GTGCCTGACCCTGATTGACA |
| 41 | TTCGTCTTGCATGGCACTCT | ATGGGCGAGCTCACTTTTGA |
| 42 | ACCCCTGTTCAACTGGTCAA | AGGTCACCAACACGGTACAC |
| 46 | TCCCTTTCCAACTACGTCGC | AAGTTCCCTTGAGGACAGCG |
| Actin^a^ | AGGCCGTTCTGTCCCTCTAC | CAGTAAGGTCACGACCAGCA |

^a^*Actin* (accession no. AB698859)
