# Supplementary material for: Proteomic Analysis of Differentially Accumulated Proteins in Cucumber (Cucumis sativus) Fruit Peel in Response to Pre-storage Cold Acclimation
Source: Front Plant Sci. 2018 Jan 18;8:2167. doi: 10.3389/fpls.2017.02167 (PMC5778441; doi:10.3389/fpls.2017.02167)
Supplement: Supplementary file 5 [file SupplementaryFigureLegends.docx]

Captions for Figure S1 and S2

**Figure S1** **| Two-dimensional electrophoresis maps from different replicates.** This figure shows the replicates that correspond to Figure 2. “NA”, Total protein from the non-acclimated fruit; “PsCA”, Total protein de novo from fruit treated with 3 d of PsCA; “Control”, Total protein from the control (12 d in cold storage). Non-acclimation (NA) samples show the proteomic profile prior to PsCA treatment. Control fruit were directly placed at 5^o^C. “Rep”, replicate.

**FIGURE S2 | Effects of pre-storage cold acclimation (PsCA) on time-dependent transcript accumulation of representative differentially accumulated proteins in cucumber.** The relative transcript accumulation was evaluated by quantitative real-time PCR using gene-specific primers (see Table S1). Control fruit were directly placed at 5^o^C. PsCA treatment was first incubated at 10^o^C for 3 d and then stored at 5^o^C. The **transcript accumulation** data were all normalized to 100 % (1.0) at 0d (before cold treatment) . Data are presented as means ± standard errors (n = 3).
