# Supplementary figures and images for: Proteomic Analysis of Differentially Accumulated Proteins in Cucumber (Cucumis sativus) Fruit Peel in Response to Pre-storage Cold Acclimation
Source: Front Plant Sci. 2018 Jan 18;8:2167. doi: 10.3389/fpls.2017.02167 (PMC5778441; doi:10.3389/fpls.2017.02167)

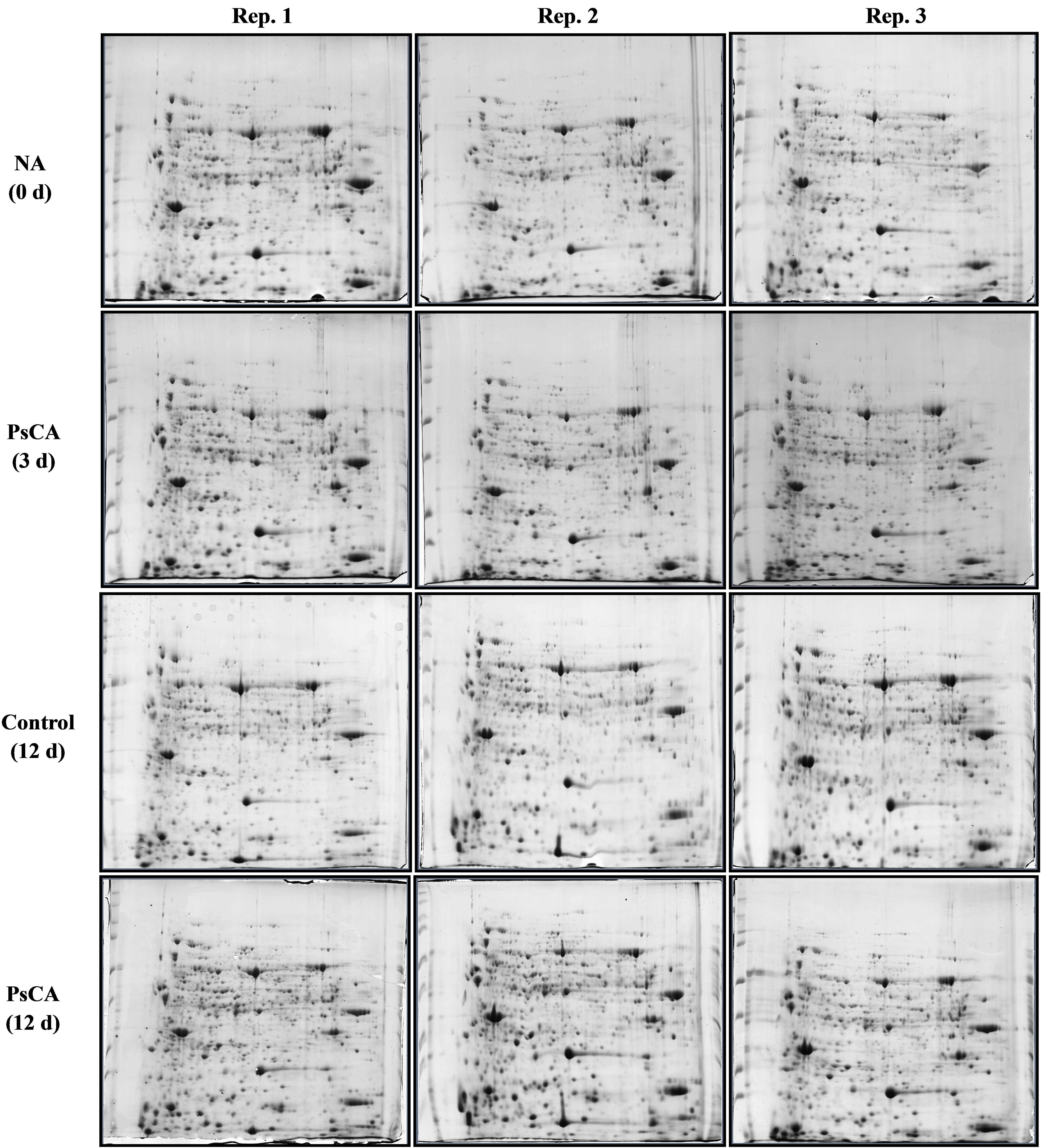

Supplement: Supplementary file 6 [file Image1.JPEG]

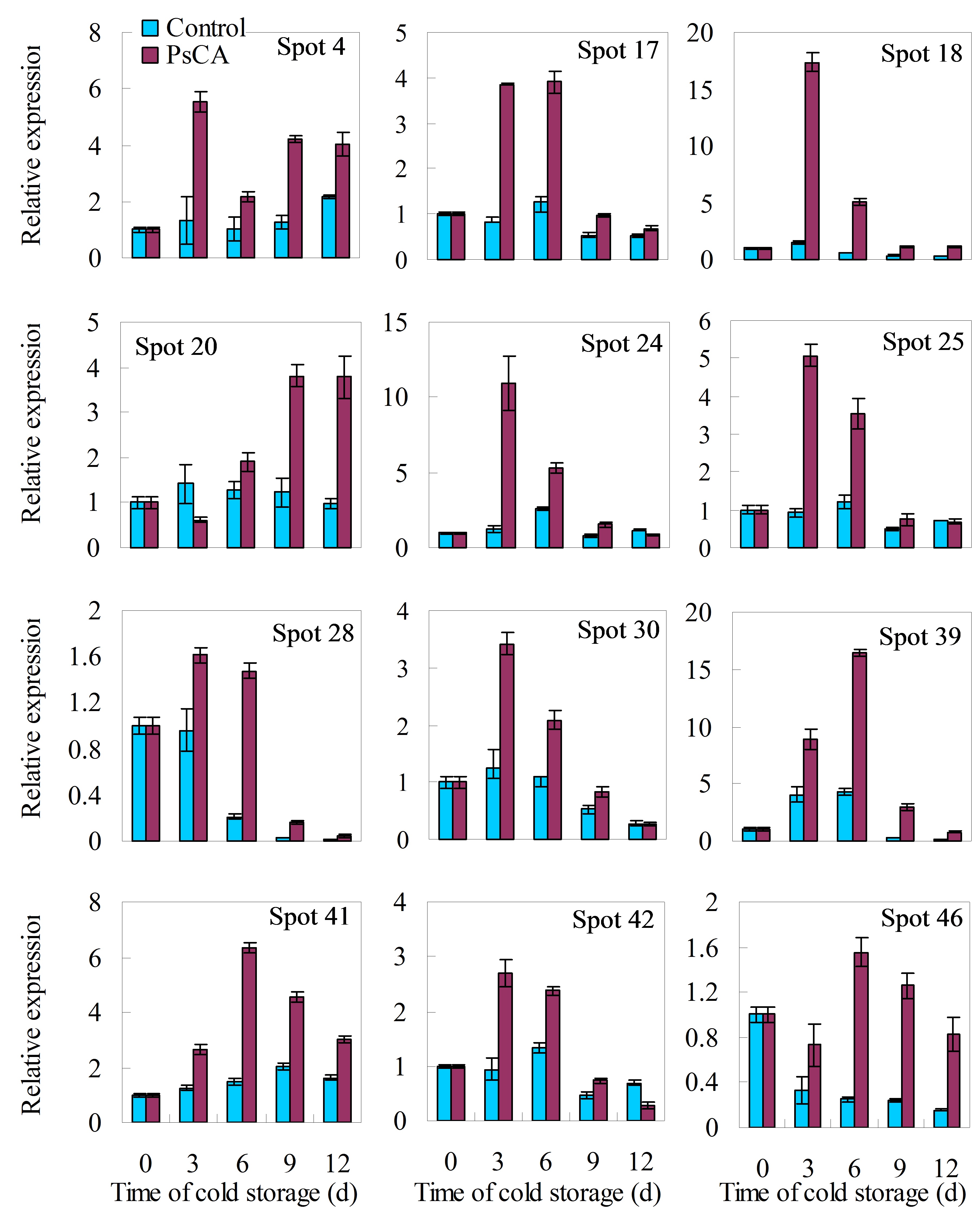

Supplement: Supplementary file 7 [file Image2.JPEG]
